# Body Mass Index and Risk of Pancreatic Cancer in a Chinese Population

**DOI:** 10.1371/journal.pone.0085149

**Published:** 2014-01-15

**Authors:** Seema Untawale, Andrew O. Odegaard, Woon-Puay Koh, Ai Zhen Jin, Jian-Min Yuan, Kristin E. Anderson

**Affiliations:** 1 Division of Epidemiology and Community Health, University of Minnesota School of Public Health, Minneapolis, Minnesota, United States of America; 2 Duke-NUS Graduate Medical School Singapore, Singapore, Republic of Singapore; 3 National Registry of Diseases Office, Health Promotion Board, Singapore, Republic of Singapore; 4 University of Pittsburgh Cancer Institute, Division of Cancer Control and Population Sciences, Pittsburgh, Pennsylvania, United States of America; 5 Tacoma-Pierce County Health Department, Tacoma, Washington, United States of America; 6 Saw Swee Hock School of Public Health, National University of Singapore, Singapore, Republic of Singapore; 7 Department of Epidemiology, University of Pittsburgh Graduate School of Public Health, Pittsburgh, Pennsylvania, United States of America; 8 Masonic Cancer Center, University of Minnesota, Minneapolis, Minnesota, United States of America; Kagoshima University Graduate School of Medical and Dental Sciences, Japan

## Abstract

Few studies have examined the association between body mass index (BMI: kg/m^2^) and pancreatic cancer risk in Asian populations. We examined this relationship in 51,251 Chinese men and women aged 45–74 who enrolled between 1993 and 1998 in the population based, prospective Singapore Chinese Health Study. Data were collected through in-person interviews. By December 31, 2011, 194 cohort participants had developed pancreatic cancer. A Cox proportional hazards model was used to estimate hazard ratios (HR) and their 95% confidence intervals (95% CI). We hypothesized the association between BMI and pancreatic cancer risk may vary by smoking status (ever v. never) and there was evidence for this as the interaction between BMI and smoking status was significant (p = 0.018). Among ever smokers, being classified as underweight (BMI <18.5 kg/m^2^), was associated with a significantly elevated risk of pancreatic cancer relative to smokers with a BMI of 21.5–24.4 kg/m^2^ (HR = 1.99, 95% CI  =  1.03–3.84). This association was strengthened after exclusion of the first three years of follow-up time. Among never smokers, there was no association between BMI and pancreatic cancer risk. However, after excluding pancreatic cancer cases and person-years in the first three years of follow-up, never smokers with a BMI ≥ 27.5 kg/m^2^ showed a suggestive increased risk of pancreatic cancer relative to never smokers with a BMI of 21.5–24.4 kg/m^2^ (HR  =  1.75, 95% CI  =  0.93–3.3). In conclusion, Singaporean Chinese who were underweight with a history of smoking had an increased risk of developing pancreatic cancer, whereas there was no significant association between BMI and pancreatic cancer in never smokers.

## Introduction

Pancreatic cancer kills more than 250,000 people each year worldwide. The malignancy is very aggressive; the mortality/morbidity ratio is 0.99 to 1 [1]–[3]. The prognosis of pancreatic cancer is extremely poor; most patients die from the malignancy within four to six months after diagnosis [2].

Despite a large number of studies on the etiology of pancreatic cancer, few consistent risk factors have been identified [4]. Only a few studies have examined the relationship between body mass index (BMI: kg/m^2^) and pancreatic cancer risk in Asian populations, and the findings have been inconsistent [5]–[8]. Previous epidemiological studies have reported increased risk of pancreatic cancer among overweight and obese individuals [8]–[10]. Most studies [10]–[13] have been conducted in Western populations that differ from Asian populations in which the majority are relatively lean when classifying by the WHO standard [23].

The generalizability of the Western findings to Asian populations is uncertain [14]. Continental Asian populations have a higher body fat percentage for a given BMI compared to Caucasians and accordingly, the consequences of obesity relevant to cancer risk may manifest at lower levels of BMI among Asians [7], [13]. In addition, dietary and other lifestyle factors in Asians differ substantially from their Western counterparts [15]. These dietary/lifestyle factors could affect the associations between BMI and incidence of pancreatic cancer. The present study is an examination of BMI as a risk factor for pancreatic cancer among participants in the Singapore Chinese Health Study; a population–based prospective cohort investigation with over 63,000 Chinese men and women in Singapore.

## Materials and Methods

### Ethics Statement

The institutional review boards at the National University of Singapore, the University of Minnesota, and the University of Pittsburgh approved this study. Written informed consent was obtained from all participants.

### The Study Population

The design of the Singapore Chinese Health Study has been previously described [14]. Briefly, the cohort was drawn from men and women, aged 45 to 74 years at enrollment, who belonged to two major dialect groups of Chinese (Hokkien and Cantonese) in Singapore. Between April 1993 and December 1998, 63,257 individuals completed an in-person interview that included questions on demographics, educational attainment, height, weight, use of tobacco and alcohol, usual physical activity, menstrual and reproductive history (women only), medical history, family history of cancer and a 165-item food frequency section assessing usual dietary intake of the previous year.

For this study we excluded 1936 subjects of the original 63,257 participants with a history of invasive cancer at baseline (except non-melanoma skin cancer or superficial, papillary bladder cancer), because they did not meet study inclusion criteria and we excluded an additional 10,070 participants who were missing measures of either, or both, height and weight. Hence, the present study included 51, 251 participants. Participants excluded due to missing BMI (N = 10,070) were not materially different from participants included in the analysis in the distributions of noted demographic and lifestyle characteristics.

### Exposure Assessment

Self-reported height and weight were collected at the baseline interview. BMI was calculated as weight (kg) divided by height squared (m^2^). Self-report of body weight has been shown to be highly valid across many populations, as well as specifically in Asians [16]. Age was defined as age in years at the time of the baseline examination. Education was categorized into no formal education, primary school, and secondary school or above. Cigarette smoking was classified into never smoker, light, and heavy smoker as described previously [17]. The “heavy” smokers were those who started to smoke before 15 years of age and smoked 13 or more cigarettes per day; all remaining ever smokers were defined as light smokers. For purposes of this study, we examined the association between “light” and ‘heavy” smoking relative to never smoking in relation to incident pancreatic cancer. Because we found no significant difference in association (see Results) we combined them into the category “ever” smokers. “Never smokers” were defined as those who have smoked less than 100 cigarettes per lifetime or without a history of smoking. We did not include tobacco chewing or other forms of tobacco in the cohort as there was only negligible use (<0.1%). A history of physician-diagnosed diabetes (yes vs. no) was included as well.

A semi-quantitative food-frequency questionnaire specifically developed for this population to assess 165 commonly consumed food items was administered during the baseline interview assessing usual dietary intake of the previous year. The questionnaire has subsequently been validated against a series of 24-hour dietary recall interviews [15], as well as selected biomarker studies [18]–[19]. Dietary patterns were derived for this study population by using principal component analysis as described previously [20] and adjusted for overall dietary pattern, including red meat. A vegetable, fruit, and soy rich pattern of diet was assessed. Alcohol was divided into none, light-moderate, and heavy drinkers based on sex (women: 0, 1–7 drinks/wk, >7 drinks/wk; men: 0, 1–14 drink/wk, >14 drinks/wk). Physical activity was assessed by using eight continuous categories ranging from never to 31 hours or more in an average week spent doing strenuous sports (e.g., jogging, bicycling on hills, tennis, squash, swimming laps, or aerobics), vigorous work (e.g., moving heavy furniture, loading or unloading trucks, shoveling, or equivalent manual labor), and moderate activities (e.g., brisk walking, bowling, bicycling on level ground, tai chi, and chi king). Usual sleep duration was assessed by asking participants the following question, “On the average, during the last year, how many hours in a day did you sleep?” Response categories were 5 hours or less, 6 hours, 7 hours, 8 hours, 9 hours, and 10 hours or more.

### Case Ascertainment

Identification of incident pancreatic cancer cases and deaths among cohort members was accomplished by record-linkage analysis of the cohort database with respective databases from the population-based Singapore Cancer Registry and the Singapore Registry of Births and Deaths. As of December 31, 2011, 194 members of this study had developed pancreatic cancer. The nationwide cancer registry has been in place since 1968 and has been shown to be comprehensive in its recording of cancer cases [21] among cohort members.

### Statistical Analysis

Study participants were initially grouped into eight categories of BMI, as reported at the baseline interview (<18.5 kg/m^2^, 18.5–19.9 kg/m^2^, 20.0–21.4 kg/m^2^, 21.5–22.9 kg/m^2^, 23.0–24.4 kg/m^2^, 24.5–25.9 kg/m^2^, 26.0–27.4 kg/m^2^, ≥27.5 kg/m^2^). These categories were created to allow for a detailed examination of the association between BMI and pancreatic cancer based on the distribution of BMI in the study population, with the consideration of BMI cut points recommended by the World Health Organization (WHO) working group for Asian populations (BMI <18.5 kg/m^2^  =  underweight, 18.5–22.9 kg/m^2^ =  normal weight, 23.0–27.4 kg/m^2^ =  overweight, ≥27.5 kg/m^2^  =  obese) [22]. We collapsed these categories further based upon similar age and sex standardized cancer rates (<18.5 kg/m^2^, 18.5–21.4 kg/m^2^, 21.5–24.4 kg/m^2^, 24.5–27.4 kg/m^2^, ≥27.5 kg/m^2^) and those who smoked cigarettes were grouped into four BMI categories (<18.5 kg/m^2^, 18.5–21.4 kg/m^2^, 21.5–24.4 kg/m^2^, ≥24.5), to provide statistical stability in the highest BMI category since there was only one case in the category of BMI ≥27.5 kg/m^2^. This approach improved the precision of the estimates, and allowed for more detailed examination through interaction and stratification, but did not alter the conclusion.

Proportional hazards (Cox) regression methods were used to estimate multivariate-adjusted hazard ratios (HR), together with the corresponding 95% confidence intervals (95% CI), and p-values of pancreatic cancer incidence by BMI group. In the primary Cox regression model, age was categorized as years at the baseline examination (<50 years, 50–54 years, 55–59 years, 60–64 years, and ≥65 years). Other variables included were sex, year of interview (1993–95 and 1996–98), dialect (Hokkien vs. Cantonese), level of education (no formal schooling, primary school, secondary school or above), baseline physician diagnosed diabetes mellitus (no, yes), smoking (no, yes), alcohol intake (none, light-moderate, heavy drinkers), vigorous work or strenuous physical activity (≥1.5 hours/week vs. <1.5 hours/week), moderate physical activity (≥2 hours/week vs. <2 hours/week), sleep (<6 hours or ≥9 hours per night vs. 6–8 hours per night), and a vegetable, fruit, and soy-rich dietary pattern score. For each subject, person-years were counted from the date of baseline interview to the date of cancer diagnosis, date of death, date of last contact or December 31, 2011, whichever occurred first.

We tested for a potential quadratic association since the data suggested that this may be a possibility. In order to test whether or not the BMI-cancer relationship was linear or quadratic (J- or U-shaped curve), linear and quadratic terms with values corresponding to the median value for each BMI category were included in the model and statistical significance evaluated using the Wald chi-square test. Because the results provided no evidence of a quadratic association, only the p-linear trend values are included in the results. The proportionality assumption was met, as indicated by the lack of significant interaction between BMI as a function of survival time in the model.

To reduce potential bias due to pre-clinical disease or cancer, illness-related weight loss, as well as accounting for the anorectic effects of smoking at the time of the baseline interview, we performed analyses with exclusion of cases diagnosed in the first three years of follow up [23]–[24]. Statistical computing was conducted using SAS statistical software version 9.1 (SAS Institute Inc., Cary, North Carolina). All p-values quoted are two-sided, and p-values <0.05 were considered statistically significant.

## Results

**Table 1** shows baseline characteristics of the 51,251 participants included in the analysis. The total person-years of follow up were 760,504. One hundred ninety-four participants developed pancreatic cancer during the follow up period. As BMI increased, mean age of the participants at baseline decreased, proportion of women increased, level of education displayed an inverse U-shaped curve, as did physical activity, diabetes was not associated with pancreatic cancer in the study, HR 1.384 (95% CI: 0.889–2.153). Additionally, the proportion of those who had ever smoked decreased, daily consumption of alcohol decreased, and dietary pattern remained primarily constant, as did energy intake and number of hours sleep. Compared to the never smokers, light and heavy smokers experienced an HR of 1.24 (95% CI  =  0.87–1.76) and 1.36 (95% CI  =  0.69–2.70), respectively. These two groups made up the category “ever” smokers in the analysis due to the lack of a significant difference in their association with pancreatic cancer risk.

**Table 1:** Participant Characteristics According to Body Mass Index (kg/m^2^) at Baseline, The Singapore Chinese Health Study.

		Body Mass Index (kg/m^2^)			
Characteristics	<18.5	18.5–21.4	21.5–24.4	24.5–27.4	≥ 27.5
N	3,850	13,705	17,730	10,650	5,316
Age (SD)	57.2 (8.2)	55.9 (8.0)	55.7 (7.8)	55.9 (7.7)	55.8 (7.9)
Sex (% Women)	51.8	54.2	54.0	53.5	59.6
Education (% Secondary)	29.7	34.0	33.2	29.8	25.3
Diabetes Mellitus (%)	4.1	6.3	9.0	11.2	14.0
Ever smoked (%)	41.3	32.5	28.8	27.6	26.6
Alcohol (% Daily)	5.8	4.3	3.1	2.5	2.3
§Dietary pattern (top 20%)	18.9	20.2	20.0	20.1	20.0
Energy intake (kcal)	1542 (546)	1586 (562)	1582 (555)	1581 (580)	1564 (613)
Sleep (hours)	7.0 (1.2)	7.0 (1.1)	7.0 (1.1)	7.0 (1.2)	7.0 (1.1)
Moderate activity (hr/week)	0.8 (2.5)	0.8 (2.6)	1.0 (2.9)	1.0 (3.0)	0.8 (2.6)
Vigorous/Strenuous Activity	0.7 (3.2)	0.8 (3.4)	0.8 (3.4)	0.9 (3.6)	0.7 (3.3)

Dietary pattern (top 20%)  =  Dietary pattern score of greatest conformity in the population to a diet rich in vegetables, fruit and soy foods.

Data for age represent mean (SD).

**Table 2** shows the association between BMI and pancreatic cancer risk in the entire cohort and subsets stratified by smoking status. There was no statistically significant association between BMI categories and risk of pancreatic cancer in never smokers. However, in ever smokers, those with a BMI <18.5 kg/m^2^ were at an increased risk of developing pancreatic cancer compared to those with a BMI between 21.5–24.4 kg/m^2^.

**Table 2:** Standardized Pancreatic Cancer rate and Hazard Ratios According to Body Mass Index (BMI), The Singapore Chinese Health Study.

BMI (kg/m^2^)	<18.5	18.5–21.4	21.5–24.4	24.5–27.4	≥ 27.5	p-trend (linear)
**Whole Population**						
No. cases/N	23/3850	55/13,705	53/17,730	47/10,650	16/5316	
*Standardized rate	41.9	27.07	19.97	29.59	20.44	0.08
HR (95% CI)	1.89 (1.15–3.09)	1.34 (0.92–1.96)	1.0	1.46 (0.99–2.17)	1.02 (0.58–1.79)	
**Never-Smoked**						
No. cases/N	8/2260	31/9255	30/12,632	31/7716	15/3903	
*Standardized rate	23.52	21.83	15.47	26.31	25.62	0.58
HR (95% CI)	1.52 (0.70–3.33)	1.47 (0.89–2.43)	1.0	1.64 (0.99–2.71)	1.57 (0.84–2.92)	
**Ever-Smoked** †						
No. cases/N	15/1590	24/4450	23/5098	17/4347		0.012
*Standardized rate	72.06	39.26	32.19	27.98		
HR (95% CI)	1.99 (1.03–3.84)	1.20 (0.68–2.13)	1.0	0.91 (0.48–1.70)		

SCHS =  Singapore Chinese Health Study.

Standardized rate = Age and sex standardized cancer rate per 100,000 person years using person year time, age & sex distributions of SCHS.

HR (95% CI)  =  Hazard Ratio; 95% confidence interval: Model adjusted for age, sex, year of enrollment, dialect, education, diabetes status,

smoking (in whole population analysis), age of initiation of smoking habits, number of cigarettes per day, years of smoking, alcohol intake, dietary pattern score,

physical activity, sleep and energy intake.

BMI categories combined for ever-smokers into ≥ 24.5 kg/m^2^_._

Because potential underlying disease or poor health of the subjects at baseline might have an impact on the risk of developing pancreatic cancer, we excluded pancreatic cancer cases and person-years that occurred within the first three years post-enrollment (**Table 3**). Never smokers with a BMI ≥27.5 kg/m^2^ showed a suggestive increased risk of pancreatic cancer relative to never smokers with a BMI of 21.5–24.4 kg/m^2^ (HR  =  1.75, 95% CI  =  0.93–3.3). In ever smokers the association in participants with a BMI <18.5 kg/m^2^ was strengthened after excluding early deaths, suggesting the results were not due to underlying confounding issues with early cases having low BMIs. There was no evidence that the association differed by sex or age.

**Table 3:** Standardized Pancreatic cancer rates and Hazard Ratios According to Body Mass Index (BMI), Excluding the First Three Years of Follow-Up, The Singapore Chinese Health Study.

BMI (kg/m^2^)	<18.5	18.5–21.4	21.5–24.4	24.5–27.4	≥ 27.5	p-trend (linear)
**Whole Population**						
No. cases/N	20/3701	45/13,364	48/17,367	38/10,408	16/5193	
*Standardized rate	36.63	22.21	18.13	23.98	20.49	0.25
HR (95% CI)	1.83 (1.08–3.09)	1.21 (0.81–1.82)	1.0	1.31 (0.85–2.0)	1.13 (0.64–2.0)	
**Never-Smoked**						
No. cases/N	5/2205	26/9109	27/12,457	25/7591	15/3831	
*Standardized rate	14.74	18.33	13.94	21.26	25.67	0.22
HR (95% CI)	1.06 (0.41–2.76)	1.36 (0.79–2.33)	1.0	1.47 (0.85–2.54)	1.75 (0.93–3.3)	
**Ever-Smoked** †						
No. cases/N	15/1496	19/4255	21/4910	14/4175		0.007
*Standardized rate	72.58	31.25	29.52	23.15		
HR (95% CI)	2.25 (1.15–4.4)	1.05 (0.56–1.96)	1.0	0.82 (0.41–1.61)		

SCHS =  Singapore Chinese Health Study.

Standardized rate = Age and sex standardized cancer rate per 100,000 person years using person year time, age & sex distributions of SCHS.

HR (95% CI)  =  Hazard Ratio; 95% confidence interval: Model adjusted for age, sex, year of enrollment, dialect, education, diabetes status,

smoking (in whole population analysis), age of initiation of smoking habits, number of cigarettes per day, years of smoking, alcohol intake, dietary pattern score,

physical activity, sleep and energy intake.

BMI categories combined for ever-smokers into ≥ 24.5 kg/m^2^_._

## Discussion

We observed a differential association between BMI and incidence of pancreatic cancer in never vs. ever smoking Chinese men and women in Singapore. In never smokers there was no evidence of any association between BMI and incident pancreatic cancer until excluding participants with potential underlying disease or poor health, when a suggestive association between obesity and incident pancreatic cancer appeared. On the other hand, underweight ever smokers with a BMI <18.5 kg/m^2^ were at an increased risk for pancreatic cancer even after accounting for potential underlying disease and poor health. A number of lifestyle and behavioral factors were evaluated as covariates and did not alter the risk of pancreatic cancer observed for BMI and smoking.

Previous reviews have concluded that an obese BMI is associated with increased pancreatic cancer risk [25]–[27]. However, data from individual studies of this topic are contradictory. Five Western and Northern European investigations plus two meta-analyses investigated the association between BMI and pancreatic cancer [3], [25], [28]–[31]. Findings ranged from what appeared to be an inverse association in women, to a 3.3-fold increase in risk in a comparison of highest to lowest BMI. Our data suggest that the association between BMI and the risk of pancreatic cancer in the Singapore population may be different from that in Western populations. The Singapore Chinese Health study provides a unique population to examine this question since the BMI range in the study population allows for the examination of low BMI <18.5 kg/m^2^ and cancer risk. Most other study populations could not do so, due to an extremely low percent of study subjects with BMI <18.5 kg/m^2^.

With respect to Asian populations, the association between BMI and pancreatic cancer has been inconsistent in the published data. Results from the Japan Collaborative Cohort pooled study [5], reported in general, no significant association between increased BMI and risk of pancreatic cancer. However, a Korean study reported a statistically significant increase in the risk of pancreatic cancer (HR  =  1.8; 95% CI: 1.14–2.86) for women with BMI ≥30 kg/m^2^, but not in men [7]. In the US Multiethnic Cohort Study, that included 51.3% of Asian-Americans, an increased risk of pancreatic cancer was found in the complete cohort; men having an increased BMI, and women having an inverse association [36]. In another Japanese study [28], a significant inverse association was found between BMI and the risk of pancreatic cancer in men (especially smokers). Compared with men having a BMI of 21–25 kg/m^2^, risk was elevated among lean men with a BMI of <21 kg/m^2^ and reduced among men with a BMI of ≥25 kg/m^2^.

A review of 21 prospective studies with a total of 8,062 pancreatic cancer cases revealed that all studies adjusted for, but did not stratify by smoking [25]. When stratified by smoking, previous studies have suggested that the positive relationship between BMI and pancreatic cancer risk might be stronger in never smokers than in ever smokers [5], [10], [30], [32]–[39]. Some studies have shown strong BMI-associated pancreatic cancer risk, as estimated from death rates, in never smokers compared with that observed in the overall population [10], [37], [40]. We saw a suggestive association of increased risk of pancreatic cancer in never smokers having BMI ≥27.5 after exclusion of the first three years of follow up.

Some inconsistencies in the literature might be due to variations in how BMI was modeled across studies and to the small number of cases in a number of studies. Additional differences between our findings and those of others could be due to either different BMI criteria being used to define obesity (e.g., Western population cut points), different adjustments of covariates, different reference categories, different classes of exposure (e.g., study- and specific quartiles) and/or different study populations. Further investigations are needed to clarify how BMI across the life span is associated with pancreatic cancer in Asian populations.

Insulin regulation may be the mechanism linking obesity and pancreatic cancer. A hyperinsulinemic state, leading to an increased bioavailability of insulin-like growth factor-I, could stimulate cell proliferation and lead to tumorigenesis [41]. An alternative mechanism for the association between BMI and pancreatic cancer may be formation of lipid peroxidation-

related DNA adducts and DNA damage [42]. The levels of these types of DNA adducts have been reported to be significantly higher in pancreatic cancer patients, and positive correlations were found between obesity and levels of lipid peroxidation [43]. A plausible mechanism for an increasing risk of pancreatic cancer with increasing BMI, insulin regulation and/ or lipid peroxidation, may not explain the cancer risk association with a lower BMI (<18.5 kg/m^2^).

Additional studies have shown that smokers typically have a lower BMI and body weight than do non-smokers [44]–, as nicotine increases metabolic rate and energy expenditure [48]–[49]. Carcinogens generated by smoking may mask the effect of increasing BMI associated with risk of pancreatic cancer. A 2007 systematic review found that increased body fatness tended to be associated with decreased risk for cancer in cohort studies [28], possibly reflecting reverse causation or lower body weight in heavy smokers.

Limitations include the use of self-reported height, weight and other demographic and lifestyle data. Although the use of self-reported body weight and height could be prone to non-differential misclassification and thus lead to under-estimation of the BMI-pancreatic cancer risk association, self-report of body weight has been shown to be highly valid across many populations [14], and specifically in Asians. A review of 64 studies suggested the difference between the self-reported and objectively measured body mass index was generally null or slightly underestimated (less than 1 kg/m^2^) for those with BMI less than 30 kg/m^2^ [50] and 97% of our study population had BMI <30 kg/m^2^. Nevertheless, additional data on other measures of body habitus may complement BMI in this population and contribute to further understanding. As well, multiple assessments of relative weight may offer further insight on the topic. Furthermore, despite thorough adjustment for smoking, alcohol, dietary patterns, activity and sleep, residual confounding and unmeasured confounding need to be considered in the interpretation.

Strengths of our study include the assumption that case ascertainment was complete, given that Singapore is a small city-state where there is thorough specialized medical care available and the nationwide cancer registry has been in place since 1968 and shown to be comprehensive in its recording of cases [21]. We also controlled for suspected risk factors for pancreatic cancer. Additionally, using this prospective study, there was good follow up of members and outcome assessment, and we included adjustments for the impact of potential modifiers and confounders of the association between BMI and pancreatic cancer. The low BMI of the study population provided a unique opportunity to examine the association between a lower spectrum of BMI, i.e., <18.5 kg/m^2^, and pancreatic cancer risk, that was impossible in most other populations with high BMI.

In summary, we observed an increased risk of pancreatic cancer in ever smokers with a BMI <18.5 kg/m^2^, and a suggestive increased risk in never smokers with a BMI ≥ 27.5 kg/m^2^ in analyses that accounted for potential biases and confounding factors. Thus, the data from this study underscore the consideration of smoking status, one of the few recognized risk factors for pancreatic cancer, when examining BMI in relation to pancreatic cancer risk. Continued thorough investigation of this topic in varied populations will further increase the understanding of the relationship between BMI and pancreatic cancer risk.
